# Stanniocalcin 1 and 1,25-dihydroxyvitamin D_3_ cooperatively regulate bone mineralization by osteoblasts

**DOI:** 10.1038/s12276-024-01302-2

**Published:** 2024-09-02

**Authors:** Jung Ha Kim, Kabsun Kim, Inyoung Kim, Semun Seong, Jeong-Tae Koh, Nacksung Kim

**Affiliations:** 1 Department of Pharmacology, Chonnam National University Medical School, Gwangju, 61469 Republic of Korea; 2 Hard-Tissue Biointerface Research Center, School of Dentistry, Chonnam National University, Gwangju, 61186 Republic of Korea; 3 Department of Pharmacology and Dental Therapeutics, School of Dentistry, Chonnam National University, Gwangju, 61186 Republic of Korea

**Keywords:** Hormone receptors, Transcription

## Abstract

Stanniocalcin 1 (STC1) is a calcium- and phosphate-regulating hormone that is expressed in all tissues, including bone tissues, and is involved in calcium and phosphate homeostasis. Previously, STC1 expression was found to be increased by 1,25-dihydroxyvitamin D_3_ [1,25(OH)_2_D_3_] administration in renal proximal tubular cells. In this study, we investigated whether STC1 directly regulates osteoblast differentiation or reciprocally controls the effects of 1,25(OH)_2_D_3_ on osteoblasts to contribute to bone homeostasis. We found that STC1 inhibited osteoblast differentiation in vitro and bone morphogenetic protein 2 (BMP2)-induced ectopic bone formation in vivo. Moreover, 1,25(OH)_2_D_3_ increased STC1 expression through direct binding to the *Stc1* promoter of the vitamin D receptor (VDR). STC1 activated the 1,25(OH)_2_D_3_–VDR signaling pathway through the upregulation of VDR expression mediated by the inhibition of Akt phosphorylation in osteoblasts. STC1 further increased the effects of 1,25(OH)_2_D_3_ on receptor activator of nuclear factor-κB ligand (RANKL) secretion and inhibited osteoblast differentiation by exhibiting a positive correlation with 1,25(OH)_2_D_3_. The long-bone phenotype of transgenic mice overexpressing STC1 specifically in osteoblasts was not significantly different from that of wild-type mice. However, compared with that in the wild-type mice, 1,25(OH)_2_D_3_ administration significantly decreased bone mass in the STC1 transgenic mice. Collectively, these results suggest that STC1 negatively regulates osteoblast differentiation and bone formation; however, the inhibitory effect of STC1 on osteoblasts is transient and can be reversed under normal conditions. Nevertheless, the synergistic effect of STC1 and 1,25(OH)_2_D_3_ through 1,25(OH)_2_D_3_ administration may reduce bone mass by inhibiting osteoblast differentiation.

## Bone mineralization regulation: Stanniocalcin 1 & Vitamin D3 synergize

In the field of bone health, it’s important to understand how our bodies control bone creation. This research investigates how Stanniocalcin 1 and 1,25-dihydroxyvitamin D3 work together to affect bone mineralization by osteoblasts. The team used mouse models and cell cultures in their experiment. They found that STC1 alone can slow down osteoblast differentiation and bone creation, but this effect is stronger when 1,25-dihydroxyvitamin D3 is also present. This suggests a complex relationship that could affect bone health. They conclude that the combined regulation by STC1 and 1,25-dihydroxyvitamin D3 is a key factor in bone mineralization, providing new insights into managing bone health. This knowledge could lead to treatments for bone diseases by targeting these pathways. Future research may show how to adjust these interactions for treatment benefits, potentially improving results for those with bone health problems.

This summary was initially drafted using artificial intelligence, then revised and fact-checked by the author.

## Introduction

Stanniocalcin (STC) was first identified in fish as a secreted phosphoglycoprotein^1–3^. In fish, STC controls calcium and phosphate homeostasis by inhibiting calcium uptake in the gills and intestines and stimulating phosphate reabsorption in the kidneys^1–3^. The STC1 gene, encoding a homolog of the fish STC gene, has been isolated from humans, mice, and other mammals^4,5^. Mammalian STC1 shares approximately 80% amino acid similarity with fish STC in terms of the number and location of Cys residues. The similarity between fish STC and mammalian STC1 indicates that mammalian STC1 regulates calcium and phosphate homeostasis in the gut and kidneys^6^. STC1 is expressed in various adult and fetal tissues, including those in the kidneys, intestines, and bones, all of which are known to be involved in calcium and phosphate homeostasis^3–15^. In particular, cellular expression of STC1 has been noted in chondrocytes and osteoblasts during murine development^16^. Based on the pattern of STC1 expression during growth and development, several studies have suggested that STC1 plays a role in regulating calcium and phosphate levels in the epithelium of the choroid plexus, in addition to regulating mesenchymal-epithelial interactions, endothelial activation, angiogenesis, and chondrocyte differentiation^15,17–19^.

Bone tissues are mineralized throughout an individual’s life. The complex physiological process of biomineralization is regulated by the balance between stimulators and inhibitors of biomineralization at the autocrine/paracrine and systemic levels^20,21^. Local biomineralization is initiated by the synthesis of phosphatases, such as phosphatase orphan 1 (PHOSPHO1) and tissue-nonspecific alkaline phosphatase (TNAP), by bone-forming osteoblasts^20,22,23^. PHOSPHO1 and TNAP serve as stimulators of biomineralization by providing phosphate, which is necessary for the nucleation of crystal growth within matrix vesicles, and hydrolyzing pyrophosphate (PPi) to produce inorganic orthophosphate (Pi) in the microenvironment of osteoblasts^21,24,25^. However, inorganic pyrophosphate, a strong inhibitor of mineralization, is also produced by osteoblasts^21,25^. In the adult human body, approximately 85% of the total phosphorus is stored in bones as hydroxyapatite (calcium–phosphate) crystals. As phosphate is essential for skeletal mineralization, both hyper- and hypophosphatemia can lead to impaired skeletal mineralization, which is characteristic of rickets and osteomalacia^26^.

STC1 is expressed in osteoblasts and plays an important role in phosphate homeostasis. Therefore, STC1 may be is involved in local biomineralization by osteoblasts. Despite the efforts of several studies, whether STC1 functionally participates in osteoblast development remains largely unknown. Contrasting results have been reported on the role of STC1 during osteoblast differentiation in vitro. Yoshiko et al. reported that STC1 downregulation reduced osteoblast differentiation, whereas recombinant STC1 treatment potentiated osteoblast differentiation in rat calvarial cells^6^. In contrast, Johnston et al. reported that compared with those from wild-type mice, calvarial cells from transgenic mice overexpressing STC1 exhibited delayed osteoblast differentiation^27^. Furthermore, in some studies, *Stc1*-null mice did not exhibit an overt skeletal phenotype, whereas hSTC1-hyperstimulated mice exhibited skeletal deficiencies, including calvarial bone hypoplasia, suggesting that STC1 plays a sharply contrasting role in biomineralization^16,27^. We therefore assessed whether STC1 is functionally involved in local biomineralization by osteoblasts.

## Materials and Methods

### Cell culture

Primary osteoblast precursor cells were isolated from neonatal mouse calvariae. The initial treatment involved the exposure of the calvariae to 0.1% collagenase (Life Technologies, Carlsbad, CA, USA) and 0.2% dispase II (Roche Diagnostics GmbH, Mannheim, Germany) at 37 °C for 10 min, after which the first set of cells was discarded. Subsequently, primary osteoblast precursor cells were obtained from the calvariae through four sequential enzymatic digestions, each lasting 10 min. For induction of osteoblast differentiation, the cells were cultured in osteogenic medium (OGM) containing 100 ng/mL BMP2 (Busan, Korea), 50 μg/mL ascorbic acid (Sigma, St. Louis, MO, USA), and 10 mM β-glycerophosphate (Sigma) for 3 or 6 days. After 3 days of culture, alkaline phosphatase (ALP) activity was analyzed. Cell lysates were prepared using osteoblast lysis buffer [composed of 50 mM Tris–HCl (pH 7.4), 1% Triton X-100, 150 mM NaCl, and 1 mM EDTA]. These lysates were then incubated with a p-nitrophenyl phosphate substrate (Sigma), and ALP activity was quantified by measuring the absorbance at 405 nm using a spectrophotometer. Moreover, after 6 days of culture, Alizarin Red staining was performed. The cultured cells were fixed with 70% ethanol and treated with 40 mM Alizarin Red solution (pH 4.2) for 10 min. The stained cultures were visualized using CanoScan 4400 F (Canon, Inc., Japan). For quantification of substrate calcification, Alizarin Red was extracted using 10% cetylpyridinium chloride solution. The concentration of Alizarin Red was then determined by measuring the absorbance at 570 nm using a spectrophotometer.

For induction of osteoclast differentiation, cells from the bone marrow and osteoblasts were cocultured in α-MEM supplemented with 10% FBS, 1,25-dihydroxyvitamin D_3_ [1,25(OH)_2_D_3_, 10^−8^ M] (Sigma), and prostaglandin E_2_ (PGE_2_, 10^−7^ M, Sigma). The cultured cells were fixed with 3.7% formalin, stained with tartrate-resistant acid phosphatase (TRAP), and observed under a Leica DMIRB microscope equipped with an N Plan 10 × 0.25 numerical aperture objective lens (Leica Microsystems, Wetzlar, Germany).

### Retroviral gene transduction

Plat E, a packaging cell line, was transfected with retroviral vectors using FuGENE 6 (Promega, Madison, WI, USA) following the manufacturer’s instructions. After 48 h of transfection, viral supernatants were collected from the culture media. Osteoblasts were seeded onto 48-well or 6-well plates 24 h before infection. Osteoblasts were then incubated with the viral supernatants for 6 h in the presence of 10 μg/mL polybrene (Sigma).

### Quantitative real-time polymerase chain reaction (qPCR)

qPCR analyses were conducted in triplicate using the Rotor-Gene Q system (Qiagen, GmbH, Hilden, Germany) and the SYBR Green PCR Master Mix (Qiagen). For analysis of target gene expression, the expression data were normalized to the level of an endogenous control, specifically *Gapdh*. The relative quantitative value for each target gene compared with the calibrator for that target was expressed as 2^−(Ct–Cc)^, where Ct and Cc represent the mean threshold cycle differences after normalization to *Gapdh*. The relative expression levels of the samples are presented in a semilog plot. The sequences of primers used were as follows: *Gapdh*, 5ʹ-TGA CCA CAG TCC ATG CCA TCA CTG-3ʹ and 5ʹ-CAG GAG ACA ACC TGG TCC TCA GTG-3ʹ; *Runx2*, 5ʹ-CCC AGC CAC CTT TAC CTA CA-3ʹ and 5ʹ-CAG CGT CAA CAC CAT CAT TC-3ʹ; *Alpl* (ALP), 5ʹ-CAA GGA TAT CGA CGT GAT CAT G-3ʹ and 5ʹ-GTC AGT CAG GTT GTT CCG ATT C-3ʹ; *Ibsp* (BSP), 5ʹ-GGA AGA GGA GAC TTC AAA CGA AG-3ʹ and 5ʹ-CAT CCA CTT CTG CTT CTT CGT TC-3ʹ; *Bglap* (osteocalcin), 5ʹ-ATG AGG ACC CTC TCT CTG CTC AC-3ʹ and 5ʹ-CCA TAC TGG TTT GAT AGC TCG TC-3ʹ; *Vdr*, 5ʹ-GGA TCT GTG GAG TGT GTG GAG ACC-3ʹ and 5ʹ-CTT CAT CAT GCC AAT GTC CAC GCA G-3ʹ; and *Tnfsf11* (RANKL), 5ʹ-CCT GAG ACT CCA TGA AAA CGC-3ʹ and 5ʹ-TCG CTG GGC CAC ATC CAA CCA TGA-3ʹ.

### Chromatin immunoprecipitation (ChIP)

ChIP was performed using an EZ-ChIP™ kit (Millipore, Burlington, MA, USA) following the manufacturer’s instructions. In brief, osteoblasts were cultured with or without vitamin D_3._ The protein and DNA of the cells were crosslinked. The DNA was subsequently fragmented by sonication. Protein–DNA complexes were then immunoprecipitated using either IgG or an anti-vitamin D receptor (VDR) antibody (Santa Cruz Biotechnology, Dallas, TX, USA). Subsequently, the protein–DNA complexes were eluted, and reverse crosslinking was performed. DNA purification was performed using spin columns provided in the EZ-ChIP™ kit (Millipore). The purified DNA was subsequently amplified by PCR. The primer sequences targeting the VDR-binding sites within the *Stc1* promoter region were as follows: 5ʹ-TAT TTC AGG GTG GAA AGA TG-3ʹ and 5ʹ-ATT CAC CTGA AAC ATC ATG C-3ʹ.

### Short interfering RNA (siRNA) transfection

Dharmacon (Lafayette, CO, USA) provided control, *Stc1*, and *Vdr* siRNAs, which were then transfected into osteoblasts using Lipofectamine RNAiMAX (Thermo Fisher Scientific, Waltham, MA, USA), following the manufacturer’s protocol.

### Western blotting

Cultured cells were harvested using lysis buffer containing 50 mM Tris–HCl (pH 8.0), 150 mM NaCl, 1 mM EDTA, 0.5% Nonidet P-40, 1 mM PMSF, and a protease inhibitor cocktail. After centrifugation at 13,000 × *g* for 15 min at 4 °C, protein concentrations were determined using the BCA Protein Assay Kit (Pierce, Rockford, USA). Equal amounts of protein were then separated by sodium dodecyl sulfate-polyacrylamide gel electrophoresis and transferred to a polyvinylidene fluoride membrane (Millipore). The membranes were subsequently incubated with the appropriate primary antibodies. After washes and incubation with suitable horseradish peroxidase-linked secondary antibodies (Abcam, Cambridge, UK), signals were visualized using an enhanced chemiluminescence reagent and the Azure c300 chemiluminescent western blot imaging system (Azure Biosystems, Inc., Dublin, CA, USA). The primary antibodies used were as follows: FLAG and actin (Sigma) and p-Akt, Akt, p-JNK, JNK, p-p38, p38, p-Erk, and Erk (Cell Signaling Technology, Danvers, MA, USA).

### Microcomputed tomography (μCT)

The specimens were scanned and analyzed using the SkyScan 1172 system (SkyScan, Kontich, Belgium). Scanning was performed at 50 kV and 201 μA with a 0.5 mm aluminum filter, resulting in a resolution of 11 μm per pixel. Images were acquired at intervals of 0.7° over an angular range of 180°. The raw images were reconstructed into serial cross-sections, and femoral morphometric parameters were assessed using the following specific software tools: NRecon 1.4 for image reconstruction, CTAn for data analysis, and Ant 2.4 for 3-dimensional model visualization (all provided by SkyScan).

### Histological analysis

Tibiae were harvested from STC1 transgenic mice and their wild-type littermates. The bones were fixed with 4% paraformaldehyde overnight and then decalcified in 5.5% EDTA for 3 weeks at 4 °C. After decalcification, the tibiae were dehydrated and embedded in paraffin blocks. Longitudinal Section (4 μm thick) were cut from the paraffin blocks. These sections were then deparaffinized using xylene. TRAP staining and hematoxylin-eosin (H&E) staining were performed to identify osteoclasts and osteoblasts, respectively.

### Ectopic bone formation

Four-week-old male mice [Institute of Cancer Research (ICR)] were anesthetized by intraperitoneal injection of 0.1% avertin (Sigma). In the ectopic bone formation model, 1.2 µg of BMP2-treated collagen sponges were combined with the vehicle, STC1 (Cloud-Clone Corp., Wuhan, China), 1,25(OH)_2_D_3_ (0.04 µg), or STC1 (3 µg) + 1,25(OH)_2_D_3_ (0.04 µg). These constructs were individually implanted under the dorsal skin on both sides of the anesthetized mice. After 4 weeks, all mice were euthanized with CO_2_ to evaluate bone generation.

### Mice

All animal experiments were approved by the Chonnam National University Medical School Research Institutional Animal Care and Use Committee (IACUC; CNU IACUC-H-2021-60). The animal experiments were performed in accordance with the approved guidelines. Six-week-old healthy ICR male mice were randomly assigned to 3 groups. The mice were injected intraperitoneally with vehicle or 1,25(OH)_2_D_3_ (10 µg/kg or 100 µg/kg) for 3 consecutive days. The mice were euthanized the next day.

### Statistical analyses

Statistical analyses were conducted using a two-tailed Student’s *t*-test for 3 independent samples or analysis of variance (ANOVA) followed by post hoc Tukey’s HSD test for multiple group comparisons. All the data are reported as the means ± standard deviations (SDs). p values less than 0.05 were considered to indicate statistical significance.

## Results

### STC1 inhibits osteoblast mineralization in mouse calvarial cell cultures

**Fig. 1:**
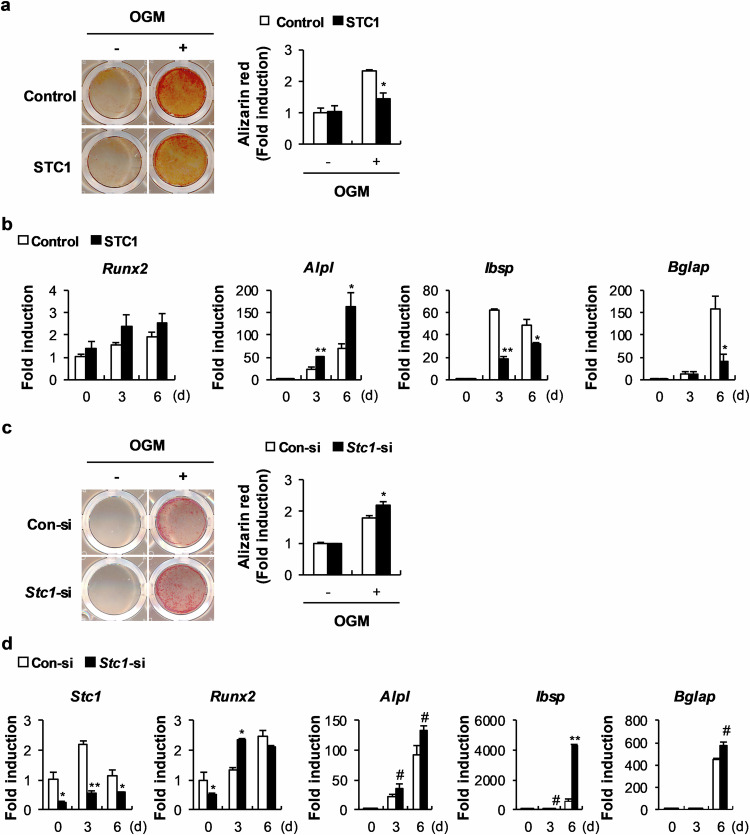
Stanniocalcin 1 (STC1) negatively regulates bone morphogenetic protein 2 (BMP2)-induced osteoblast differentiation and function. **a**, **b** Osteoblast precursors transduced with pMX-FIG (control) or pMX-STC1 were cultured in osteogenic medium (OGM). **a** The cultured cells were stained with Alizarin Red (left) and quantified via extraction (right) (*n* = 3). **b** Relative mRNA expression levels of the indicated genes were determined by real-time PCR (*n* = 3). **c**, **d** Osteoblast precursors transfected with negative control siRNA (Con-si) or *Stc1* siRNA (*Stc1*-si) were cultured in OGM. **c** The cultured cells were stained with Alizarin Red (left) and quantified via extraction (right) (*n* = 3). **d** Relative mRNA expression levels of the indicated genes were determined by real-time PCR (*n* = 3). The data are presented as the means ± SDs of triplicate samples. #*p* < 0.05, *p < 0.01, **p < 0.001 vs. the control.

To clarify the specific effects of STC1 on osteoblasts, we retrovirally overexpressed STC1 in primary mouse osteoblast precursor cells, and the cells were cultured in OGM. STC1 overexpression significantly reduced nodule formation, as shown by Alizarin Red staining (Fig. 1a). An assessment of the changes in the mRNA levels of osteoblast-related genes due to STC1 overexpression revealed a strong decrease in the expression levels of *Ibsp* and *Bglap*, which are relatively late marker genes for osteoblast differentiation (Fig. 1b). To confirm the effects of STC1 on osteoblasts, we downregulated *Stc1* expression in primary mouse osteoblast precursor cells using siRNA. As expected, *Stc1* downregulation significantly increased nodule formation and elevated the mRNA expression levels of *Ibsp* and *Bglap* (Fig. 1c, d). In contrast, both *Stc1* overexpression and downregulation increased the expression of *Alpl*, an early marker gene for osteoblast differentiation. Although the exact reason remains unclear, these results, together with the finding that STC1 did not affect ALP activation (data not shown), indicate that the change in *Alpl* expression is not due to a specific effect of STC1. Collectively, these results indicate that STC1 reduces osteoblast mineralization.

### STC1 functionally promotes the effect of 1,25(OH)_2_D_3_ on osteoblasts

**Fig. 2:**
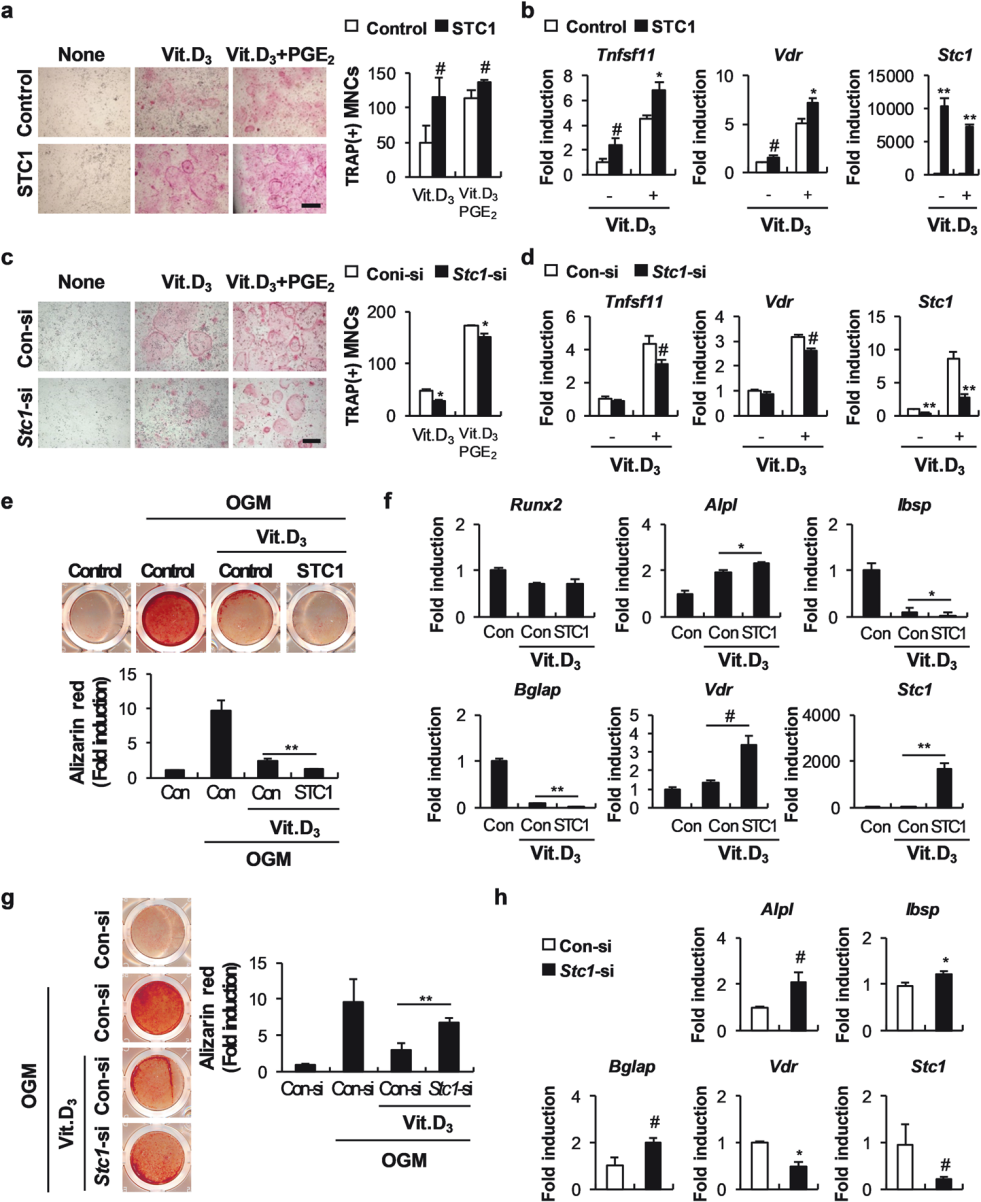
STC1 increases 1,25(OH)_2_D_3_ (Vit. D_3_)-mediated osteoclast formation. **a** Osteoblast precursors transduced with pMX-FIG (control) or pMX-STC1 were cocultured with bone marrow cells in the presence or absence of Vit. D_3_ (10^−8^ M) or Vit. D_3_ + prostaglandin E2 (PGE_2,_ 10^−7^ M). The cultured cells were stained with tartrate-resistant acid phosphatase (TRAP, left), and TRAP-positive MNCs were quantified (right) (*n* = 3). Bar, 200 µm. **b** Osteoblast precursors transduced with pMX-FIG (control) or pMX-STC1 were cultured in the presence or absence of Vit. D_3_ (10^−8^ M). The relative mRNA expression levels of the indicated genes were determined by real-time PCR (*n* = 3). **c** Osteoblast precursors transfected with Con-si or *Stc1*-si were cocultured with bone marrow cells in the presence or absence of Vit. D_3_ (10^−8^ M) or Vit. D_3_ (10^−8^ M) + PGE_2_ (10^−7^ M). The cultured cells were stained with TRAP (left), and TRAP-positive MNCs were quantified (right) (*n* = 3). Bar, 200 µm. **d** Osteoblast precursors transfected with Con-si or *Stc1*-si were cultured in the presence or absence of Vit. D_3_ (10^−8^ M). The relative mRNA expression levels of the indicated genes were determined by real-time PCR (*n* = 3). **e**, **f** Osteoblast precursors transduced with pMX-FIG (control) or pMX-STC1 were cultured in OGM in the presence or absence of Vit. D_3_ (10^−8^ M). **e** The cultured cells were stained with Alizarin Red (upper) and quantified via extraction (lower) (*n* = 3). **f** Relative mRNA expression levels of the indicated genes were determined by real-time PCR (*n* = 3). **g**, **h** Osteoblast precursors transfected with Con-si or *Stc1*-si were cultured in OGM in the presence or absence of Vit. D_3_ (10^−8^ M). **g** The cultured cells were stained with Alizarin Red (left) and quantified via extraction (right) (*n* = 3). **h** Relative mRNA expression levels of the indicated genes were determined by real-time PCR (*n* = 3). The data are presented as the means ± SDs of triplicate samples. #*p* < 0.05, *p < 0.01, **p < 0.001 vs. the control.

The hormone 1,25(OH)_2_D_3_ is essential for calcium and phosphate homeostasis and bone mineralization. Low vitamin D levels are associated with inadequate bone mass or remodeling, indicating that adequate vitamin D levels are essential for bone health^28^. Interestingly, we previously reported that similar to STC1, 1,25(OH)_2_D_3_ directly reduced osteoblast mineralization (Supplementary Fig. 1)^29^. Therefore, we investigated whether STC1 regulates the effects of 1,25(OH)_2_D_3_ on osteoblasts. 1,25(OH)_2_D_3_ supports osteoclast differentiation by inducing RANKL (*Tnfsf11*), an essential cytokine for osteoclast differentiation derived from osteoblasts_._ To determine whether STC1 affects the supportive effects of 1,25(OH)_2_D_3_ on osteoclast differentiation, we cocultured STC1-overexpressing osteoblasts and osteoclast precursor cells and treated them with 1,25(OH)_2_D_3_ or 1,25(OH)_2_D_3_ and prostaglandin E2 (PGE_2_). Osteoclast formation was successfully induced by 1,25(OH)_2_D_3_ and PGE_2_ and was further increased in the STC1-overexpressing osteoblasts (Fig. 2a). Moreover, RANKL expression induced by 1,25(OH)_2_D_3_ in osteoblasts was significantly promoted by STC1 overexpression (Fig. 2b). In contrast, STC1 downregulation in osteoblasts attenuated both osteoclast formation and RANKL expression induced by 1,25(OH)_2_D_3_ (Fig. 2c, d). These results suggest that STC1 indirectly supports osteoclast differentiation by increasing the ability of 1,25(OH)_2_D_3_ to induce RANKL. Next, we assessed whether STC1 is also involved in the 1,25(OH)_2_D_3_-mediated suppression of osteoblast mineralization. The inhibitory effect of 1,25(OH)_2_D_3_ on osteoblast mineralization was further promoted by STC1 overexpression (Fig. 2e). Moreover, the expression levels of *Ibsp* and *Bglap* were further reduced (Fig. 2f). In contrast, the effect of 1,25(OH)_2_D_3_ on osteoblast mineralization was blocked by STC1 downregulation (Fig. 2g, h). These results suggest that STC1 increases the ability of 1,25(OH)_2_D_3_ to regulate osteoblast mineralization and osteoblast-dependent osteoclast differentiation.

### STC1 promotes the effect of 1,25(OH)_2_D_3_ by increasing VDR expression

**Fig. 3:**
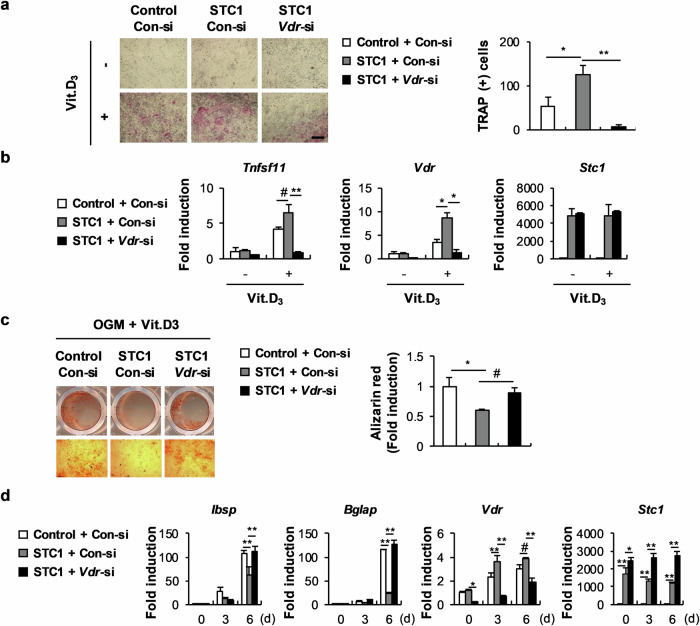
The VDR is responsible for STC1 activity in osteoblasts and osteoblast-mediated osteoclast differentiation. **a** Osteoblast precursors transduced with pMX-FIG or pMX-STC1 were transfected with Con-si or *Vdr*-si and then cocultured with bone marrow cells in the presence or absence of Vit. D_3_ (10^−8^ M). The cultured cells were stained with TRAP (left), and TRAP-positive MNCs were quantified (right) (*n* = 3). Bar, 200 µm. **b** Osteoblast precursors transduced with pMX-FIG or pMX-STC1 were transfected with Con-si or *Vdr*-si and then cultured in the presence or absence of Vit. D_3_ (10^−8^ M). The relative mRNA expression levels of the indicated genes were determined by real-time PCR (*n* = 3). **c**, **d** Osteoblast precursors transduced with pMX-FIG or pMX-STC1 were transfected with Con-si or *Vdr*-si and then cultured in OGM in the presence or absence of Vit. D_3_ (10^−8^ M). **c** The cultured cells were stained with Alizarin Red (left) and quantified via extraction (right) (*n* = 3). **d** Relative mRNA expression levels of the indicated genes were determined by real-time PCR (*n* = 3). The data are presented as the means ± SDs of triplicate samples. #*p* < 0.05, *p < 0.01, **p < 0.001 vs. the control.

As shown in Fig. 2, a positive correlation was found between *Stc1* and *Vdr* expression levels in osteoblasts stimulated with 1,25(OH)_2_D_3_. Therefore, we investigated whether the positive regulation of 1,25(OH)_2_D_3_ function by STC1 in osteoblasts is related to *Vdr* upregulation. The ability of STC1-overexpressing osteoblasts to increase osteoclast formation by increasing 1,25(OH)_2_D_3_-mediated RANKL expression was dramatically inhibited by *Vdr* downregulation (Fig. 3a, b). In addition, the strengthening effects of STC1 on the 1,25(OH)_2_D_3_-mediated suppression of osteoblast mineralization and the expression of late marker genes for osteoblast differentiation were attenuated by *Vdr* downregulation (Fig. 3c, d). These results collectively indicate that STC1 participates in the 1,25(OH)_2_D_3_–VDR signaling pathway by increasing *Vdr* expression in osteoblasts.

### The upregulation of *Vdr* expression by STC1 is associated with the inhibition of Akt phosphorylation in osteoblasts

**Fig. 4:**
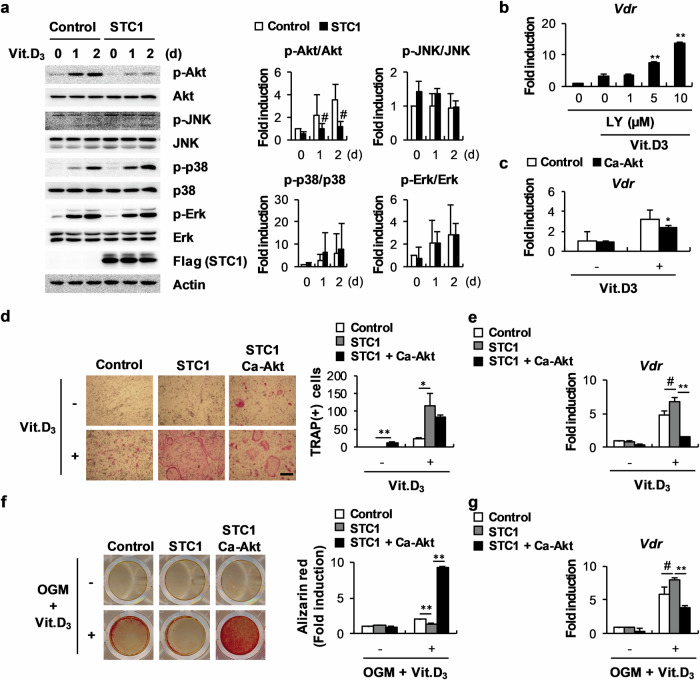
STC-induced *Vdr* expression is associated with the inhibition of Akt phosphorylation. **a** Osteoblast precursors transduced with pMX-FIG or pMX-STC1 were cultured in the presence or absence of Vit. D_3_ (10^−8^ M) for the indicated times. Cell lysates were subjected to western blot analysis with the indicated antibodies (*n* = 3). **b** Relative mRNA expression levels of *Vdr* in osteoblast precursors cultured in the presence or absence of Vit. D_3_ (10^−8^ M) in the presence of various doses of LY294002, as indicated, were determined by real-time PCR (*n* = 3). **c** Relative mRNA expression levels of *Vdr* in osteoblast precursors transduced with pMX-FIG or pMX-Ca-Akt and cultured in the presence or absence of Vit. D_3_ (10^−8^ M) were determined by real-time PCR (*n* = 3). **d** Osteoblast precursors transduced with pMX-FIG, pMX-STC1, or pMX-STC1 + pMX-Ca-Akt were cocultured with bone marrow cells in the presence or absence of Vit. D_3_ (10^−8^ M). The cultured cells were stained with TRAP (left), and TRAP-positive MNCs were quantified (right) (*n* = 3). Bar, 200 µm. **e** Osteoblast precursors transduced with pMX-FIG, pMX-STC1, or pMX-STC1 + pMX-Ca-Akt were cultured in the presence or absence of Vit. D_3_ (10^−8^ M). Relative mRNA expression levels of *Vdr* were determined by real-time PCR (*n* = 3). **f**, **g** Osteoblast precursors transduced with pMX-FIG, pMX-STC1, or pMX-STC1 + pMX-Ca-Akt were cultured in the presence or absence of OGM + Vit. D_3_ (10^−8^ M). **f** The cultured cells were stained with Alizarin Red (left) and quantified via extraction (right) (*n* = 3). **g** Relative mRNA expression levels of *Vdr* were determined by real-time PCR (*n* = 3). The data are presented as the means ± SDs of triplicate samples. #*p* < 0.05, *p < 0.01, **p < 0.001 vs. the control.

Next, we assessed the mechanism by which STC1 regulates VDR expression in osteoblasts. By examining whether STC1 can affect the signaling pathways regulated by 1,25(OH)_2_D_3_ in osteoblasts, we found that STC1 strongly inhibited Akt phosphorylation (Fig. 4a). The correlation between Akt phosphorylation and *Vdr* expression was revealed by the finding that treatment with the PI-3 kinase inhibitor LY294002, which inhibits Akt phosphorylation, increased *Vdr* expression in a dose-dependent manner, while the overexpression of a constitutively active form of Akt (Ca-Akt) significantly suppressed *Vdr* expression (Fig. 4b, c). The stimulatory effects of STC1 on 1,25(OH)_2_D_3_-mediated *Vdr* upregulation and osteoclast differentiation were abolished by Ca-Akt overexpression (Fig. 4d, e). Moreover, osteoblast mineralization and *Vdr* expression, which were further regulated by STC1 in the presence of 1,25(OH)_2_D_3_, were restored by Ca-Akt overexpression (Fig. 4f, g). In fact, the unique functions of Akt in osteoblasts other than regulating *Vdr* expression make it difficult to demonstrate that STC1 supports osteoclast differentiation and inhibits osteoblast differentiation through *Vdr* upregulation in an Akt phosphorylation inhibition-dependent manner. Nevertheless, these results suggest that STC1 contributes to 1,25(OH)_2_D_3_-mediated *Vdr* expression by suppressing Akt phosphorylation.

### 1,25(OH)_2_D_3_ upregulates *Stc1* expression in osteoblasts

**Fig. 5:**
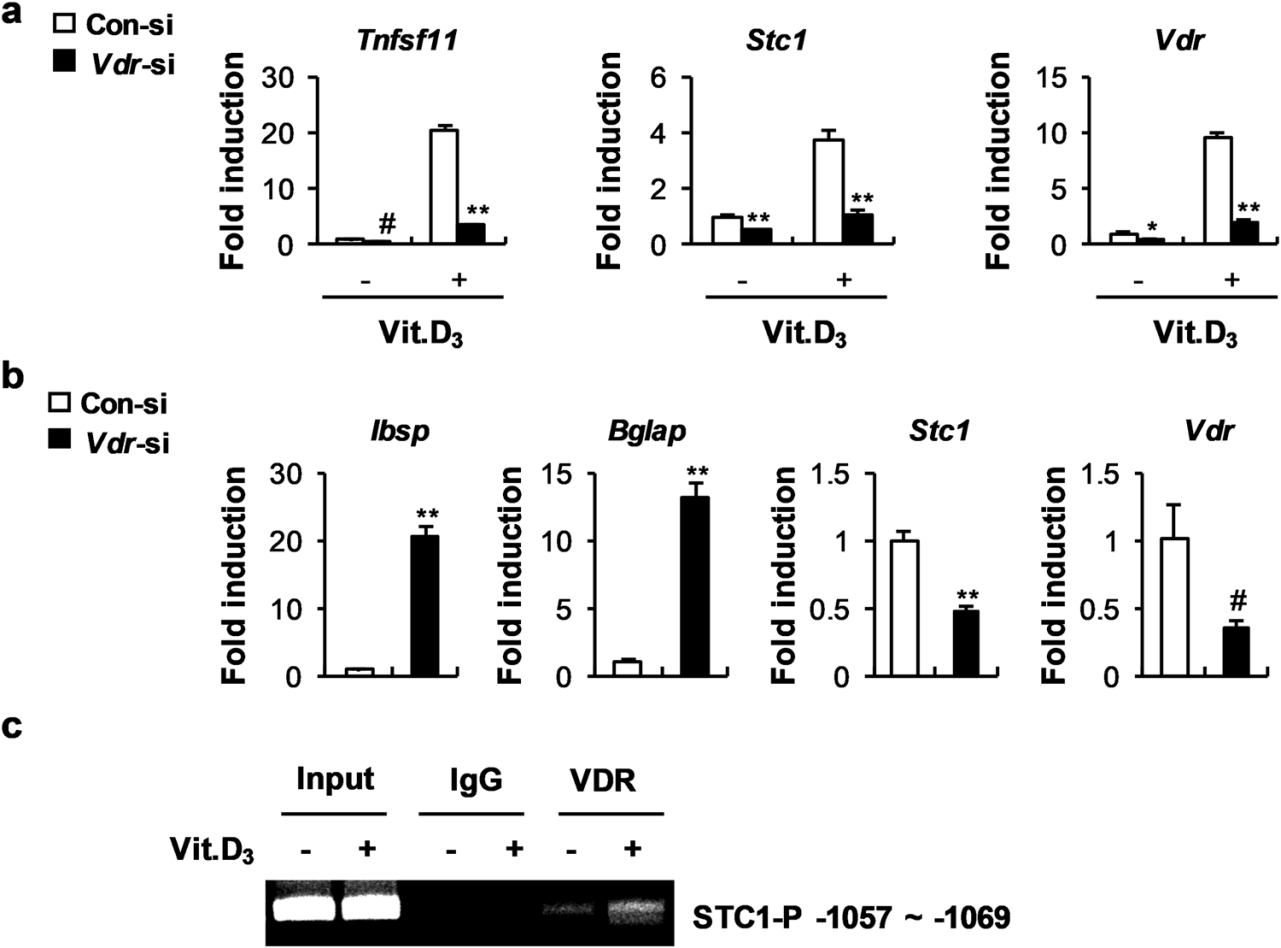
Vit. D_3_ positively regulates STC1 expression through transcriptional regulation. **a** Osteoblast precursors transfected with Con-si or *Vdr*-si were cultured in the presence or absence of Vit. D_3_ (10^−8^ M). The relative mRNA expression levels of the indicated genes were determined by real-time PCR (*n* = 3). **b** Osteoblast precursors were transfected with Con-si or *Vdr*-si and then cultured in OGM + Vit. D_3_ (10^−8^ M). The relative mRNA expression levels of the indicated genes were determined by real-time PCR (*n* = 3). **c** Immunoprecipitation was performed using anti-VDR antibodies or IgG as the negative control. Precipitated DNA was subjected to PCR with primers targeting the VDR binding site (−1057 to −1069). The data are presented as the means ± SDs of triplicate samples. #*p* < 0.05, *p < 0.01, **p < 0.001 vs. the control.

STC1 expression was reported to be upregulated by 1,25(OH)_2_D_3_ in renal proximal tubular cells^30^. We found that the expression of *Vdr* and *Stc1* was similarly increased by 1,25(OH)_2_D_3_ stimulation in osteoblasts (Fig. 2). Therefore, we examined whether VDR can reciprocally regulate *Stc1* expression. *Vdr* downregulation in the presence of 1,25(OH)_2_D_3_ reduced *Stc1* expression in both osteoblast precursor cells and differentiating osteoblasts despite 1,25(OH)_2_D_3_ stimulation (Fig. 5a, b). To further determine whether VDR regulates the transcriptional activity of *Stc1*, we performed a ChIP assay. Analysis of the *Stc1* promoter revealed a putative VDR-binding site located at positions −1057 to −1069 in the Stc1 promoter. A ChIP assay performed to confirm whether this region is a functional vitamin D response element revealed that VDR binds at positions −1057 to −1069 in the *Stc1* promoter and that the binding of VDR to the *Stc1* promoter was increased in the osteoblasts stimulated with 1,25(OH)_2_D_3_ (Fig. 5c). Collectively, these results indicate that VDR upregulates the mRNA expression of *Stc1* by binding to the *Stc1* promoter to increase the activity of the 1,25(OH)_2_D_3_–VDR signaling pathway in osteoblasts.

### STC1 attenuates bone formation in vivo

**Fig. 6:**
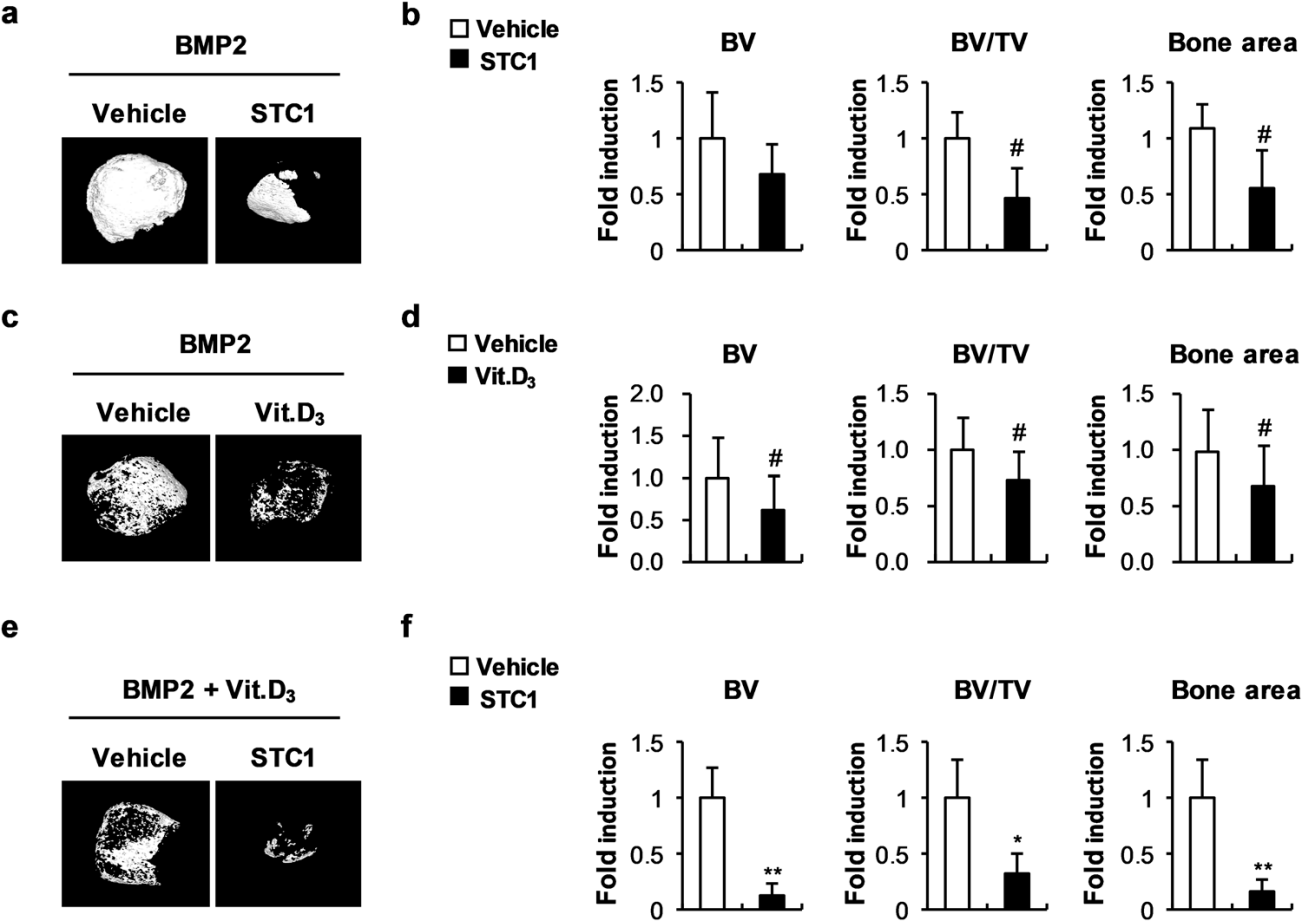
STC1 and Vit. D_3_ inhibit ectopic bone formation in vivo. **a**, **b** Collagen sponges soaked with BMP2 (1.2 µg) with or without STC1 (3 µg) were subcutaneously implanted on the top of the dorsal back. Ectopic bones were biopsied and subjected to µCT analyses. **a** Representative 3D images of ectopic bones analyzed by µCT. **b** Bone volume (BV), bone volume/tissue volume (BV/TV), and bone area were determined by µCT (*n* = 6). **c**, **d** Collagen sponges soaked with BMP2 (1.2 µg) with or without Vit. D_3_ (0.04 µg) were subcutaneously implanted on the top of the dorsal back. Ectopic bones were biopsied and subjected to µCT analyses.0 **c** Representative 3D images of ectopic bones analyzed by µCT. **d** BV, BV/TV, and bone area were determined by µCT (*n* = 12). **e**, **f** Collagen sponges soaked with BMP2 (1.2 µg) and Vit. D_3_ (0.04 µg) with or without STC1 (3 µg) were subcutaneously implanted on the top of the dorsal back. Ectopic bones were biopsied and subjected to µCT analyses. **e** Representative 3D images of ectopic bones analyzed by µCT. **f** BV, BV/TV, and bone area were determined by µCT (*n* = 6). The data are presented as the means ± SDs. #*p* < 0.05, *p < 0.01, **p < 0.001 vs. the control.

To further assess the role of STC1 in local mineralization in vivo, we first determined the effect of the recombinant STC1 protein on osteoblast differentiation. Treatment with recombinant STC1 protein during osteoblast differentiation suppressed the expression of *Ibsp* and *Bglap* (Supplementary Fig. 2a). This inhibitory effect of the recombinant STC1 protein was similar to that of stimulation with 1,25(OH)_2_D_3_ during osteoblast differentiation (Supplementary Fig. 2b). To assess the effect of STC1 on BMP2-induced ectopic bone formation, we dorsally implanted BMP2- and PBS-soaked collagen sponges on one side and BMP2- and STC1-soaked collagen sponges on the other side in a subcutaneous pocket in mice. The BMP2- and STC1-soaked sponges exhibited less BMP2-induced ectopic bone formation than the BMP2- and PBS-soaked sponges (Fig. 6a, b). Similarly, the BMP2- and 1,25(OH)_2_D_3_-soaked sponges resulted in reduced ectopic bone formation mediated by BMP2 (Fig. 6c, d). The inhibitory effect of 1,25(OH)_2_D_3_ on BMP2-induced ectopic bone formation was further increased by the addition of recombinant STC1 protein (Fig. 6e, f). These results indicate that STC1 and 1,25(OH)_2_D_3_ synergistically inhibit local mineralization in vivo.

### Excessive STC1 and 1,25(OH)_2_D_3_ have adverse effects on bone homeostasis

**Fig. 7:**
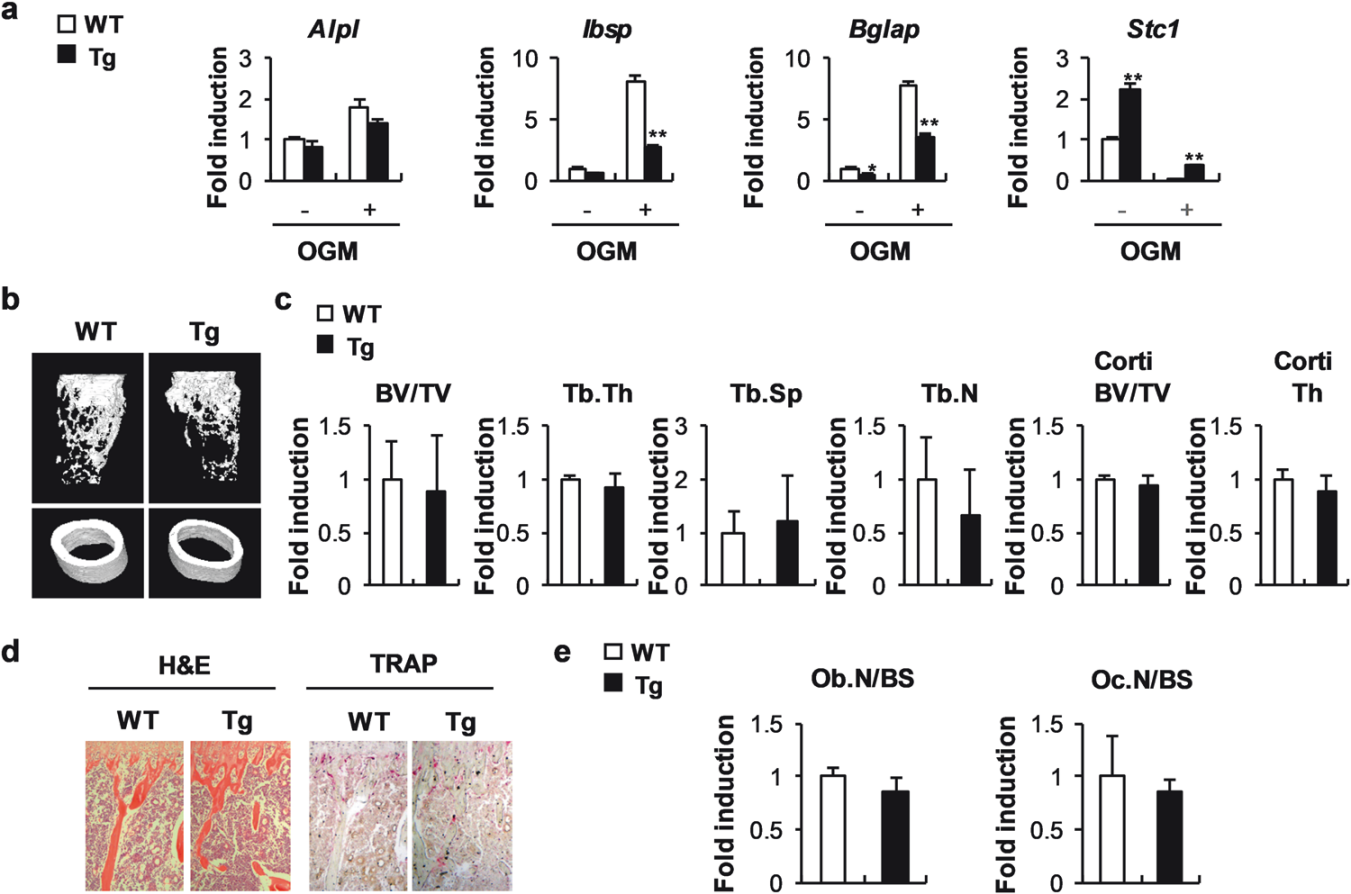
*Stc1*-overexpressing transgenic mice exhibit a mild decrease in long bone mass. **a** Bone marrow stromal cells (BMSCs) isolated from STC1 transgenic (Tg) mice or their wild-type littermates were cultured in the presence or absence of OGM. The relative mRNA expression levels of the indicated genes were determined by real-time PCR (*n* = 3). **b**–**e** Long bones of STC1 Tg mice or their wild-type littermates were subjected to µCT and histological analyses. (b) Representative 3D images of femurs from STC1 Tg mice or their wild-type littermates, as analyzed by µCT. **c** Bone volume/tissue volume (BV/TV), trabecular thickness (Tb.Th), trabecular separation (Tb.Sp), trabecular number (Tb.N), cortical bone volume/tissue volume (Corti BV/TV), and cortical thickness (Corti Th) were determined by µCT (*n* = 5 or 7). **d** H&E- and TRAP-stained images of tibiae isolated from STC1 Tg mice or their wild-type littermates. **e** Quantification of osteoblast number and osteoclast number derived from histological analyses (*n* = 5 or 7). The data are presented as the means ± SDs. #*p* < 0.05, *p < 0.01, **p < 0.001 vs. the control.

**Fig. 8:**
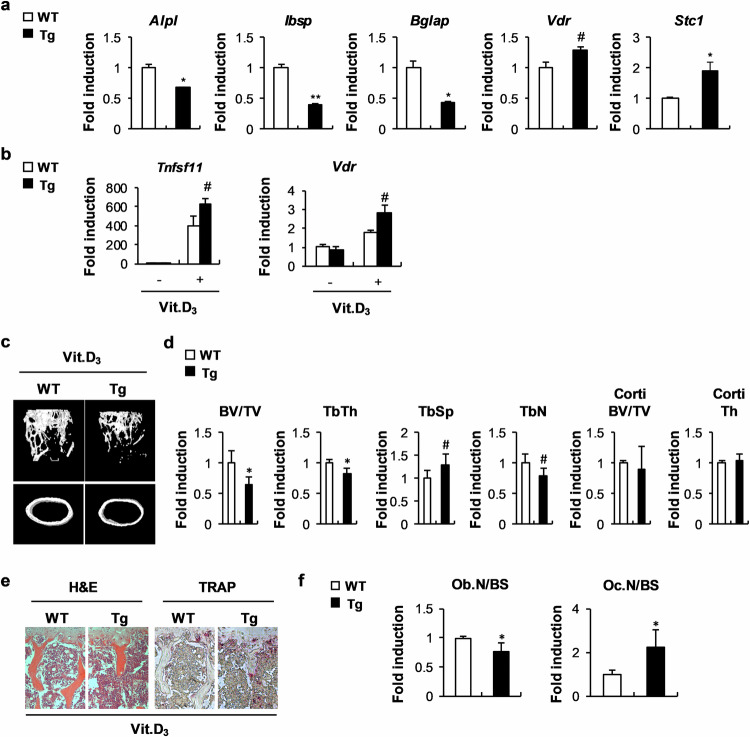
Osteoblastic STC1 overexpression reduces bone mass in the presence of Vit. D_3_ administration in vivo. **a** Osteoblasts were induced from BMSCs isolated from STC1 Tg mice or their wild-type littermates. The relative mRNA expression levels of the indicated genes were determined by real-time PCR (*n* = 3). **b** BMSCs isolated from STC1 Tg mice or their wild-type littermates were cultured in the presence or absence of Vit. D_3_ (10^−8^ M). The relative mRNA expression levels of the indicated genes were determined by real-time PCR (*n* = 3). **c**–**f** Long bones of STC1 Tg mice or their wild-type littermates were intraperitoneally injected with Vit. D_3_ and subjected to µCT and histological analyses. **c** Representative 3D images of femurs from STC1 Tg mice or their wild-type littermates intraperitoneally injected with Vit. D_3_, as analyzed by µCT. **d** Bone volume/tissue volume (BV/TV), trabecular thickness (Tb.Th), trabecular separation (Tb.Sp), trabecular number (Tb.N), cortical bone volume/tissue volume (Corti BV/TV), and cortical thickness (Corti Th) were determined by µCT (*n* = 6 or 7). **e** H&E- and TRAP-stained images of tibiae isolated from STC1 Tg mice or their wild-type littermates intraperitoneally injected with Vit.D_3_. **f** Quantification of osteoblast number and osteoclast number derived from histological analyses (*n* = 6 or 7). The data are presented as the means ± SDs. #*p* < 0.05, *p < 0.01 vs. the control.

To further elucidate the osteoblast-dependent role of STC1 in bone homeostasis in vivo, we established transgenic mice that overexpress STC1 specifically in osteoblast precursor cells. When bone marrow stromal cells (BMSCs) derived from STC1 transgenic mice were cultured in OGM, the expression of *Ibsp* and *Bglap* was significantly lower than that in the BMSCs derived from wild-type mice (Fig. 7a). Compared with the wild-type mice, the STC1 transgenic mice exhibited a slightly reduced bone mass phenotype; however, this difference was not significant (Fig. 7b, c). Similarly, the number of osteoblasts was slightly lower in the STC1 transgenic mice than in the wild-type mice (Fig. 7d, e). As negative effects of 1,25(OH)_2_D_3_ were observed during the differentiation of BMSCs into osteoblasts (Supplementary Fig. 3), we investigated whether STC1 contributes to the effect of 1,25(OH)_2_D_3_ on BMSCs derived from the STC1 transgenic mice. When BMSCs were differentiated into osteoblasts in the presence of 1,25(OH)_2_D_3_, BMSCs derived from the STC1 transgenic mice showed significantly lower expression of *Ibsp* and *Bglap* but greater expression of *Vdr* than those derived from the wild-type mice (Fig. 8a). In addition, 1,25(OH)_2_D_3_ treatment increased RANKL and *Vdr* expression in BMSCs; these expression levels were further increased in BMSCs derived from the STC1 transgenic mice (Fig. 8b). Next, we investigated whether osteoblast-specific STC1 overexpression regulates bone homeostasis when the 1,25(OH)_2_D_3_ signaling pathway was stimulated. The administration of high concentrations of 1,25(OH)_2_D_3_ to the mice significantly reduced their bone mass (Supplementary Fig. 4). The STC1 transgenic mice had a significantly lower bone mass than the wild-type mice because of decreased bone volume/total volume, trabecular thickness, and trabecular number and increased trabecular separation upon 1,25(OH)_2_D_3_ administration (Fig. 8c, d). The 1,25(OH)_2_D_3_-treated STC1 transgenic mice also exhibited decreased osteoblast numbers and increased osteoclast numbers (Fig. 8e, f). Collectively, these results suggest that the ability of STC1 in osteoblasts to support osteoclast differentiation and inhibit osteoblast differentiation may be increased by 1,25(OH)_2_D_3_, resulting in a decrease in bone mass.

## Discussion

As several studies elucidating the roles of STC1 in osteoblasts and its mechanisms in osteoblast differentiation and activity have reported controversial results, the roles of STC1 in local biomineralization by osteoblasts remain to be investigated. Yoshiko et al. reported that the overexpression and downregulation of STC1 had accelerating and inhibitory effects, respectively, on osteoblast differentiation and the mRNA expression of osteopontin and osteocalcin in mature rat osteoblast cultures but not in osteoprogenitor cell cultures^6^. These researchers also suggested that the stimulatory effect of STC1 on osteoblast differentiation was related to an increase in both sodium-dependent phosphate uptake and Pit1 gene expression in mature rat osteoblast cultures^6^. In another previous study, Johnston et al. reported that calvarial cells obtained from transgenic mice constitutively expressing human STC1 exhibited reduced viability, reduced proliferation, delayed differentiation, and reduced expression of osteocalcin^27^. These researchers also reported that Pit1 gene expression did not differ between wild-type and transgenic calvarial cell cultures^27^. However, this group found that the expression of phosphate regulators other than Pit1, including Dmp1, Sfrp4, Mepe, and Enpp1, decreased in calvarial cells derived from STC1 transgenic mice^27^. Our findings are consistent with those of previous study in the latter. We observed that STC1 negatively regulates osteoblast differentiation and the expression of late osteoblast marker genes. The overexpression of STC1 using STC1 retrovirus, recombinant STC1, and cultures of BMSCs derived from STC1 transgenic mice and the downregulation of STC1 exhibited inhibitory and accelerating effects, respectively, on osteoblast differentiation as well as *Ibsp* and *Bglap* mRNA expression. The discrepancy between the results elucidating the role of STC1 in osteoblasts, including the present results, may be due to differences in the cell type used or the STC1 source, possibly due to the cell differentiation stage-dependent dual function of STC1 in osteoblasts. Nevertheless, our results indicate that STC1 inhibits osteoblast differentiation and activity in vitro, at least in osteoblast progenitor cells, although this effect may still be controversial in mature osteoblasts.

Filvaroff et al. reported that transgenic mice expressing human STC1 under the control of a muscle-specific promoter exhibited reduced cortical bone volume and total bone volume because of their smaller size than wild-type mice^17^. In the STC1 transgenic mice, the rate of bone formation but not mineralization decreased, possibly due to reduced osteoclast activity. Similarly, contrary to the expectation that STC1 suppresses osteoblast differentiation in vitro and BMP2-induced bone formation in vivo, we confirmed that the long bones of transgenic mice expressing mouse *Stc1* under the control of an osteoblast-specific promoter did not exhibit a notably altered bone phenotype. Although *Stc1*-null mice exhibit no obvious phenotype and the long bones of STC1-hyperstimulated mice do not exhibit distinct changes in bone phenotypes, severe cranial hypoplasia has been reported in the pups of STC1-hyperstimulated mice^16,27^. As calvarial bone hypoplasia is generally indicative of decreased osteoblast progenitor proliferation and differentiation, it seems likely that stimulated STC1 delays osteoblast proliferation and differentiation during intramembranous bone development; however, the effect is not persistent. Taken together, these results suggest that STC1 is closely involved in intramembranous bone formation during development; however, it is not necessarily required for bone formation under normal conditions.

1,25(OH)_2_D_3_, an important hormone that regulates calcium absorption and bone mineralization, upregulates STC1 in renal proximal tubular cells^30^. Hence, we examined whether 1,25(OH)_2_D_3_ regulates the expression of STC1 in osteoblasts and whether STC1 can contribute to the function of 1,25(OH)_2_D_3_ in osteoblasts. The expression of STC1 in osteoblasts was strongly increased by 1,25(OH)_2_D_3_, as observed in renal proximal tubular cells. VDR, a functional nuclear receptor of 1,25(OH)_2_D_3_, directly binds to the VDR-binding region of the STC1 promoter, increasing the mRNA expression level of *Stc1*. Interestingly, STC1 increased *Vdr* expression by inhibiting Akt phosphorylation. This finding was confirmed by several results, such as increased *Vdr* expression by STC1, decreased Akt phosphorylation by STC1, and increased *Vdr* expression by an inhibitor of Akt activity. The reciprocal expression regulation noted between *Vdr* and *Stc1* suggested that 1,25(OH)_2_D_3_ and STC1 have similar functions and act cooperatively in osteoblasts. Notably, STC1 synergistically promoted the effects of 1,25(OH)_2_D_3_ on osteoblast differentiation, RANKL secretion, and bone formation both in vitro and in vivo.

Vitamin D supplements are widely recommended for bone health in the general population^31^. Vitamin D supplementation is known to improve bone health because vitamin D deficiency can cause rickets in children and osteomalacia in adults. Moreover, vitamin D indirectly stimulates bone formation by increasing calcium absorption from the intestines^31^. Several studies have reported that vitamin D contributes to bone health by directly acting on osteoblasts; however, this finding remains controversial. Studies using human osteoblasts have shown that 1,25(OH)_2_D_3_ positively regulates bone formation and mineralization^32,33^. Moreover, 1,25(OH)_2_D_3_ induces the production of ALP-positive matrix vesicles during osteoblast differentiation to increase mineralization^33,34^. In contrast, accumulating evidence indicates that 1,25(OH)_2_D_3_ negatively regulates bone formation and mineralization in murine osteoblasts^32,33^. Murine osteoblasts lacking VDR exhibit increased osteogenic potential. The hormone 1,25(OH)_2_D_3_ increases pyrophosphate (PPi) levels by inducing the expression of progressive ankylosis (ANK) and ectonucleotide pyrophosphatase phosphodiesterase (ENPP1) proteins, in addition to increasing osteopontin levels in murine osteoblasts^33^. These effects in turn decrease mineralization. The discrepancy between the results regarding the direct effects of 1,25(OH)_2_D_3_ on osteoblasts cannot be solely explained by species differences. This is because different effects of 1,25(OH)_2_D_3_ have been reported in different murine models. Specific transgenic mice with osteoblast-specific VDR overexpression exhibit increased bone formation and mineralization, while global VDR knockout mice exhibit similar bone phenotypes^32,35–37^. However, the discrepancy in the direct effects of 1,25(OH)_2_D_3_ on osteoblasts may be partly explained by the complex roles of 1,25(OH)_2_D_3_. In particular, 1,25(OH)_2_D_3_ stimulates carboxylated osteocalcin and activin A, which are established inhibitors of mineralization, and inhibits bone sialoprotein (IBSP), an established stimulator of mineralization, in human osteoblasts to prevent pathological overmineralization^33^. In this study, we demonstrated the negative effects of 1,25(OH)_2_D_3_ on osteoblast differentiation and bone formation in vitro. We found that 1,25(OH)_2_D_3_ inhibited nodule formation and the expression of osteoblast-related genes in calvarial osteoblasts. The direct effects of 1,25(OH)_2_D_3_ and STC1 on osteoblasts are very similar. Under our culture conditions, 1,25(OH)_2_D_3_ and STC1 negatively regulated osteoblast differentiation. Interestingly, both 1,25(OH)_2_D_3_ and STC1 could positively regulate osteoblast differentiation, possibly due to their complex dual functions that differ depending on the differentiation stage of osteoblasts. Therefore, in addition to reciprocal expression regulation between *Vdr* and *Stc1*, the similar effects of 1,25(OH)_2_D_3_ and STC1 on osteoblasts suggest that 1,25(OH)_2_D_3_ and STC1 complementarily or synergistically regulate osteoblast differentiation.

In a recent study, compared with the placebo, vitamin D supplements did not result in a significantly decreased risk of fractures among generally healthy midlife and older adults who were not selected for vitamin D deficiency, low bone mass, or osteoporosis^38^. In addition, among older community-dwelling women, the annual oral administration of high-dose vitamin D resulted in an increased risk of fractures^39^. In this study, 1,25(OH)_2_D_3_ significantly attenuated BMP2-induced bone formation in an ectopic bone formation experiment, which was performed to exclude the possibility that 1,25(OH)_2_D_3_ plays a role in calcium absorption from the intestines. Moreover, the administration of a high dose of 1,25(OH)_2_D_3_ dramatically reduced long bone mass. Collectively, these results suggest that 1,25(OH)_2_D_3_ has a detrimental effect on bone health by exerting a direct effect on osteoblasts under certain conditions. Another interesting result of our study is that STC1 inhibits BMP2-induced ectopic bone formation and that this effect is amplified in the presence of 1,25(OH)_2_D_3_. Furthermore, we found that the normal long bone mass of STC1 transgenic mice was significantly lower than that of wild-type mice following 1,25(OH)_2_D_3_ administration. Thus, simultaneous activation of the STC1 and 1,25(OH)_2_D_3_ signaling pathways in osteoblasts can lead to decreased bone formation.

Possible differences between species pose an obstacle to the application of our experimental results obtained from murine models to humans. However, further studies are needed to confirm the effect of the interaction between STC1 and 1,25(OH)_2_D_3_ on the differentiation and activity of human osteoblasts. These future studies will demonstrate that STC1 levels are a good indicator for determining the appropriate dose and duration of vitamin D supplementation for bone health.

## Supplementary information

Supplementary Information

## Data Availability

The data that support the findings of this study are available from the corresponding author upon reasonable request.
