# Supplementary material for: Stanniocalcin 1 and 1,25-dihydroxyvitamin D3 cooperatively regulate bone mineralization by osteoblasts
Source: Exp Mol Med. 2024 Sep 2;56(9):1991–2001. doi: 10.1038/s12276-024-01302-2 (PMC11447260; doi:10.1038/s12276-024-01302-2)
Supplement: Supplementary file 1 — Supplementary Information [file 12276_2024_1302_MOESM1_ESM.pdf]

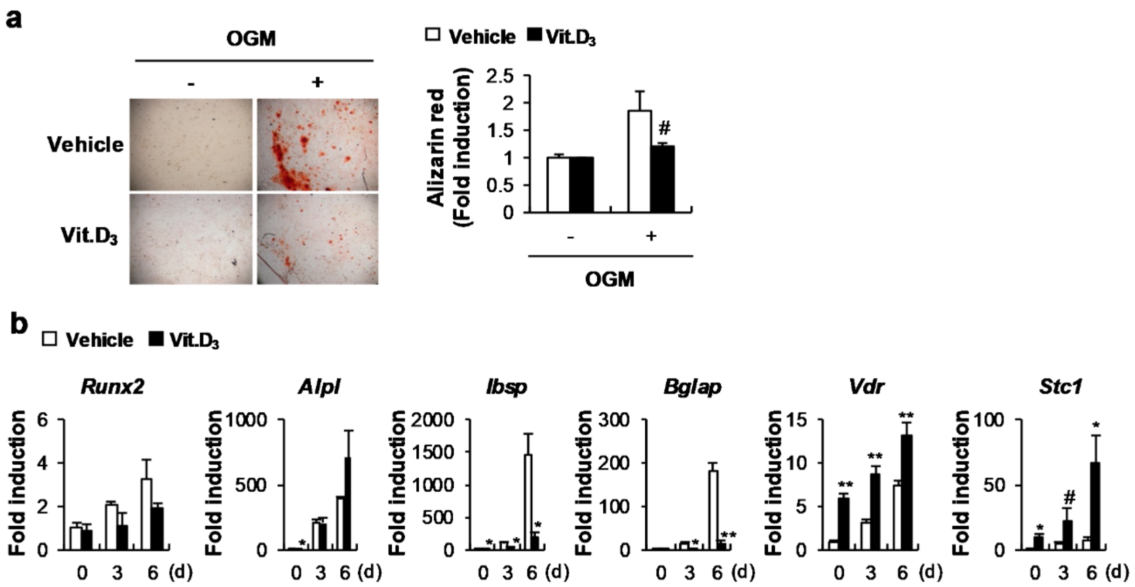

Supplementary Fig. 1. 1,25(OH)$_2$D$_3$ (Vit.D$_3$) inhibits osteoblast differentiation *in vitro*. (a, b) Osteoblast precursors were cultured in osteogenic medium (OGM) in the presence or absence of Vit.D$_3$ (10^{-8}M). (a) The cultured cells were stained with Alizarin Red (left) and quantified via extraction (right) (N = 3). Bar, 200 µm. (b) Relative mRNA expression levels of the indicated genes were determined by real-time PCR. (N = 3). Data represent the means ± SDs of triplicate samples. #p < 0.05, *p < 0.01, **p < 0.001 vs. vehicle.

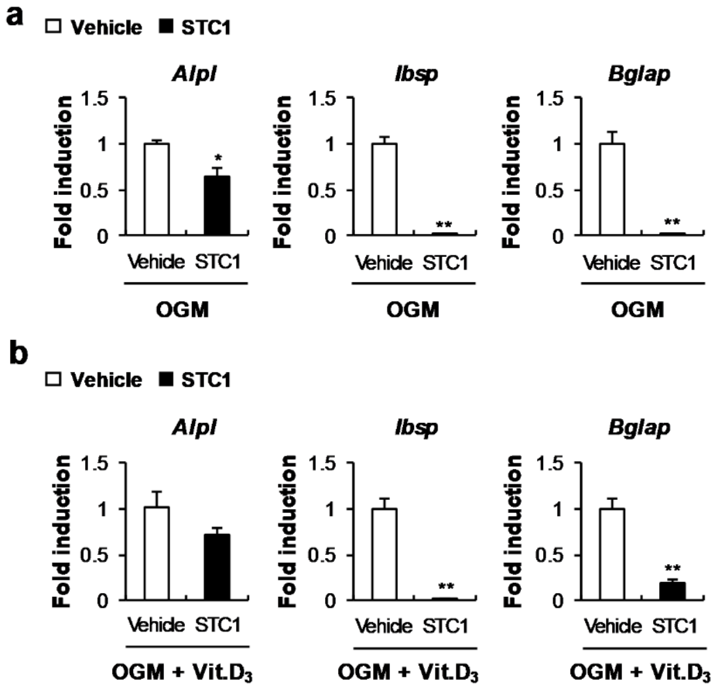

Supplementary Fig. 2. Exogenous STC1 protein negatively regulates osteoblast-related gene expression. (a) Osteoblast precursors treated with or without STC1 protein were cultured in OGM. Relative mRNA expression levels of the indicated genes were determined by real-time PCR (N = 3). (b) Osteoblast precursors treated with or without STC1 protein were cultured in OGM + Vit.D_3 (10^{-8}M). Relative mRNA expression levels of the indicated genes were determined by real-time PCR (N = 3). Data represent the means ± SDs of triplicate samples. *$p < 0.01$, **$p < 0.001$ vs. vehicle.

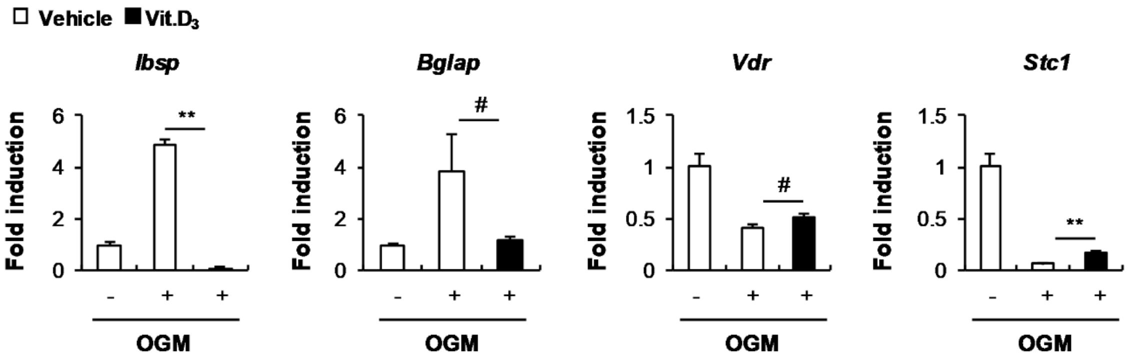

Supplementary Fig. 3. Vit.D3 suppresses osteoblast differentiation in BMSCs. Relative mRNA expression levels of the indicated genes were determined by real-time PCR (N = 3). Data represent the means ± SDs of triplicate samples. #p < 0.05, **p < 0.001 vs. vehicle.

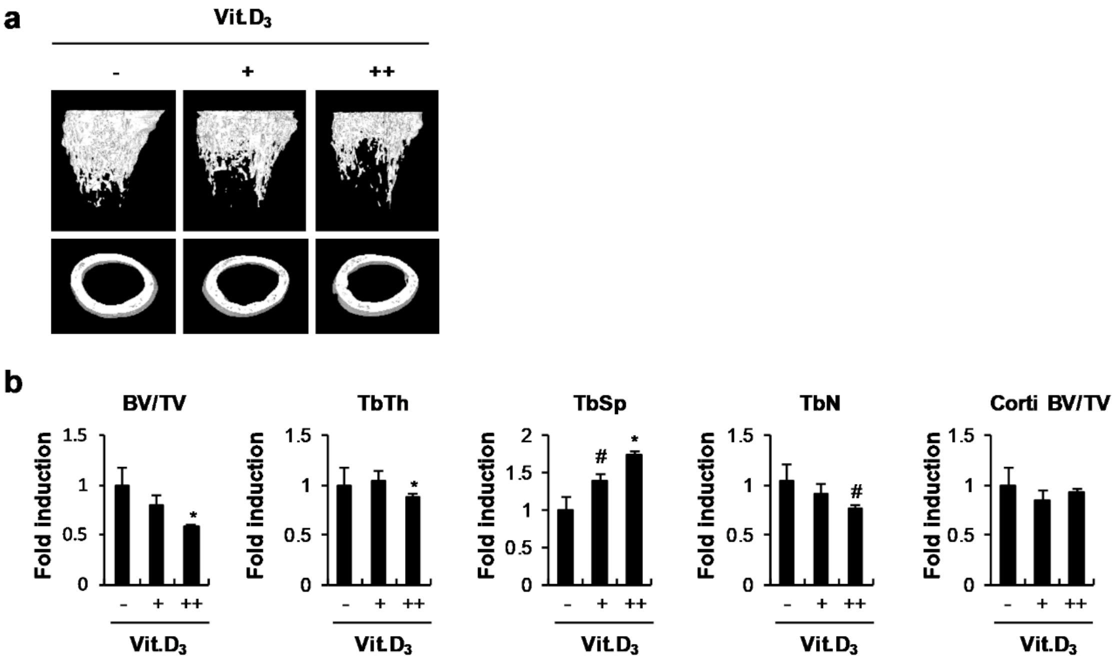

Supplementary Fig. 4. Excessive Vit.D$_3$ induces bone loss *in vivo*. (a, b) Six-week-old ICR mice were intraperitoneally injected with vehicle (control) or various doses of Vit.D$_3$ (10 µg/kg or 100 µg/kg). Femurs were isolated and subjected to µCT analyses. (a) Representative 3D images of femurs from mice intraperitoneally injected with the control or various doses of Vit.D$_3$, as analyzed by µCT. (b) Bone volume/tissue volume (BV/TV), trabecular thickness (Tb.Th), trabecular separation (Tb.Sp), trabecular number (Tb.N), and cortical bone volume/tissue volume (Corti BV/TV) were determined by µCT (N = 4). Data represent the means ± SDs. #$p < 0.05$, *$p < 0.01$ vs. control.
